# Internet or dvd for distance learning to isolated rural health professionals, what is the best approach?

**DOI:** 10.1186/s12909-017-0991-3

**Published:** 2017-09-06

**Authors:** Lanto Barthelemy Rakototiana, Rajabo, Serge Gottot

**Affiliations:** 1 Ecole Doctorale Nutrition Environnement Santé (EDNES), Université de Mahajanga, Mahajanga, Madagascar; 2 Thématique: Sante Publique, Ecole Doctorale Nutrition Environnement Santé (EDNES), Université de Mahajanga, Mahajanga, Madagascar; 3 Université Paris Diderot. Sorbonne Paris Cité, Paris, France

**Keywords:** Distance learning, Training method, Knowledge acquisition, Health professional

## Abstract

**Background:**

Distance Learning (DL) is a means to overcome the barriers that prevent health workers access to medical education and training sessions to update their knowledge. The main objective of this study is to compare the knowledge acquisition among practitioners Heads of Health Based Center (HBC) for the management of hypertension in two training modalities, one interactive, via internet (by Visio conferencing and video Conferencing), and other non-interactive, via DVD in the three regions (Miarinarivo, Moramanga and Manjakandriana) of Madagascar.

**Methods:**

This is a quasi-experimental study comparing two distance learning methodologies, one via internet (VS or VD) and the other via DVD before and after training.

Ninety-two (92) Heads of HBC participated in the training, including 56 via the Internet (24 doctors and 32 paramedics) and 36 via DVD (24 doctors and 12 paramedics).

**Results:**

According to the training mode: the mean score of knowledge of the participants was 7 (+ −2) for two terms before training. It is 14 (+ −2.5) in the internet group (VS or VD) and 15 (+ −2.7) in the DVD group after training. The difference between the two groups was not significant *p* = 0.076. For doctors, the score was 7 (+ −3.1) via internet and 8 (+ −2.3) via DVD in pre test and 14 (+ − 2.4) via internet and 16 (+ −. 2.7) via DVD in post test, the difference between the two training methods was significant (*p* = 0.008). Among the paramedics, the results are the same for both conditions, 7 (+ − 2.4 to + −3.2) in pre test and 14 (+ − 2.2 to + −2.7) in post test.

**Conclusion:**

Both training methods have improved participants’ knowledge and the DVD mode is the first choice for Heads HBC of Madagascar with the majority located in remote areas.

## Background

In medicine, progress is rapid, and it is becoming increasingly difficult for practitioners to remain well aware of the various practices regarding the management of patient medical care. Access to information is increasingly difficult for practitioners working in rural areas [1].

Traditionally, to ensure the transfer of knowledge, face-to-face training is preferred. However, due to problems related to access and cost, this is not always possible. Remote-delivery training (RDT) can mitigate these problems [2–4] by organizing training using the internet (online training, Visio conferencing (VS) and videoconferencing (VD)) or by correspondence (sending documents or digital support, such as DVDs). The main advantage of training using VS or VD is the visual contact and, in particular, the direct interaction between tutors and students (VS) or live chat (VD), which does not happen with training online or via correspondence. Training via correspondence allows a dispatch at a large scale into areas where internet access is impossible, and it offers learners greater flexibility to study the course according to their availability, including re-examining the course as much as they can, based on their needs. Training via DVD may allow a visual picture of the tutor.

In Madagascar, the management of patients’ medical care is mainly based in Health-based Centers (HBC), a first-recourse medical structure, for the majority of the population, particularly in rural areas. The function of the heads of HBC may be insured by a doctor or a paramedic (nurse or midwife). The knowledge level of these staff and their number of years in service vary according to the type of studies carried out. Consequently, there is no certainty that the latest recommendations established for the management of patient care are known and/or implemented. With the development of the internet in certain areas, and given the multiple difficulties regarding face-to-face training, it is urgent to identify the most effective remote-delivery training modalities to address these issues.

One in 4 persons over 35 years old ignores his/her arterial hypertension or is untreated [5]. The prevalence of the HTA in Antananarivo was 28.05% in 2009 and 19.10% in 2000 [6]. In Moramanga, it was estimated to be 27.6% in 2013 [7].

The number of international studies comparing the different remote-delivery training modalities is weak. None was carried out in a low-income country such as Madagascar. In the majority of studies (7/11), online training via the internet is the training modality used for the comparison, but this can be used only in locations where a permanent internet connection exists, which is not the case for the majority of health centers in Madagascar. Training using VS and VD is possible in Madagascar during the monthly regrouping of the heads of HBC in the health district office.

The goal of this study is an empirical research. The main objective was to identify the most effective remote-delivery training modalities for the training of rural doctors in Madagascar. The main objective was to compare the knowledge acquisition of the heads of HBC for the management of hypertension in two training modalities, one interactive, via internet (by Visio conferencing and video Conferencing), and other non-interactive, via DVD. The cost of each training modality and their acceptance by the practitioners was also evaluated.

## Method

This is an empirical quasi-experimental study comparing two remote-delivery training modalities, one via internet (VS and VD) and the other via DVD.

**Fig. 1:**
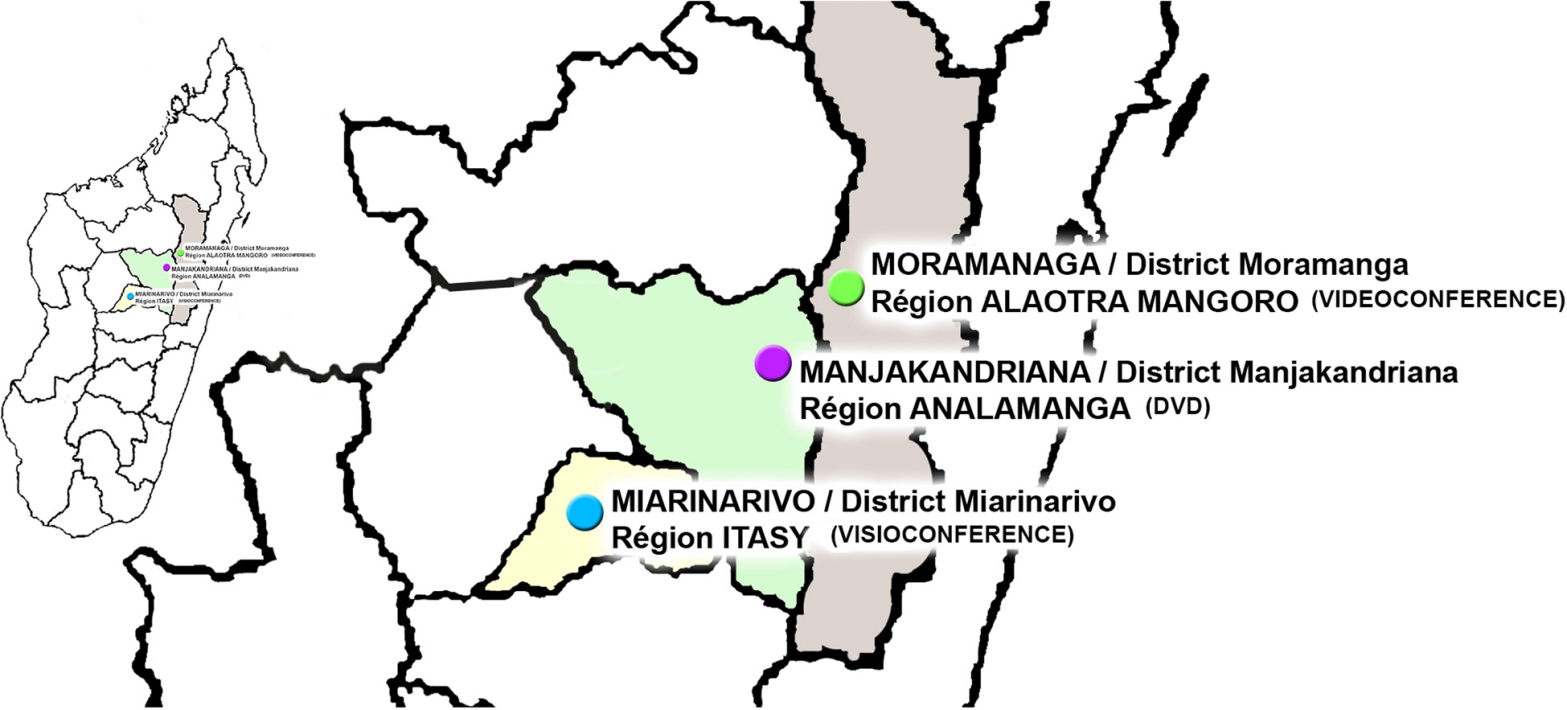
Training modality, internet (VS and VD) and per correspondence by DVD in the three areas of studies

The participants were the heads of HBC (doctors or paramedics) based in the areas Miarinarivo, Moramanga and Manjakandriana. Each area was assigned a model of training to avoid intra-area contamination and the dissemination of training content among the heads of HBC (cf. Fig. 1).

A training session where all the heads of HBC were invited was organized in each area by the District Services of Public Health (DSPH) of the High Plateaus, located at the center of the island. The training by Visio conferencing was organized live during the training session and held at the Medical Institute of Madagascar (telemedicine site of the Pan African e-network Project in Antananarivo), and the training via videoconferencing was held at the laboratory of Support to Research and Technologies Information and Communication of the Faculty of Medicine of Antananarivo. The training via VS or VD was projected on a large screen. The interaction was live with the course tutor for the VS and indirect via chat for VD. The duration of the training (including the evaluations) was two hours. The DVDs were distributed to the participants during the regrouping of the heads of the HBC. The training course was held at a doctor’s home without tutor contact.

The printed slides were distributed to the participants of the two training modalities. The training was in French, and the discussion was in Malagasy or French, according to the wishes of the participants. The contents of the training related to the synthesis of the recommendations issued by the European Society of Hypertension (ESH), the European Society of Cardiology (ESC), [8] and the French Society of Cardiology (SFC), [9] for the management of the medical care of patients with arterial hypertension. A knowledge-assessment questionnaire on the management of hypertension was developed based on questionnaires published in the literature [10–12]. The contextualization of the questionnaire was based on “Toolkit on Translating and Adapting Instruments” developed by Ligia M. Chávez. It contained 20 items, including 4 on the diagnosis, 9 on the treatment, 2 on the hygiene dietetics measure (MHD) and 5 on the follow up and the complications of hypertension. Questions were asked in the form of questions with multiple choices (QCM). It was tested on ten doctors who were not included in the study.

At the beginning of the first training course and then 1 month after the training, during the new meeting of the heads of HBC, the participants filled out the questionnaire individually.

### Evaluation of training modalities

Two tools were used to measure the rate of satisfaction:

- An individual questionnaire containing 10 items consists to know the participant’s satisfaction degree of the training modality: 3 on the appreciation of the methods and the quality of the contents of the course, 2 on the quality of the images and sound, 1 on the duration of training, 1 on the language of teaching (French or Malagasy) and 1 indicating agreement or disagreement to pay the expenses of training. The rate of satisfaction was noted on a scale from 1 to 3 (1: very satisfactory, 2: satisfying and 3: not satisfactory).
- A focus group by site. Each group was composed of 8 heads of HBC (4 doctors and 4 paramedics for Visio conferencing, 5 doctors and 3 paramedics for videoconferencing, and 6 doctors and 2 paramedics for training via DVD). Participants were included if they had at least 5 years of service as a head of HBC, were locatedless than 50 km from a road accessible to the health district year round, and agreed to take part in the focus group. An interview guideline was developed. It comprised 2 items on general appreciation, 1 on the proceeding of the training, 3 on its contents, 3 on the document distributed and 2 on possible suggestions to improve the contents and training modality.

### Costs of the evaluation

The cost of each training modality was divided into expenditures related to the investment in IT equipment and expenditures linked to the operation costs. The capital expenditures were supported by the Medical Institute of Madagascar for the VS and by the Research Laboratory in Technology Information and Communication for the VD and the recording of the DVD. The operating expenses comprised common expenditures (tutors’ fees, the knowledge-assessment questionnaire, and training support (printing of the slides) and specific expenditures for each type of training modality (cost of internet connection and manufacturing costs of the DVDs).

### Data analysis

The qualitative data were described as numbers and percentages, and the quantitative data were described as the mean (standard deviation) or median (quartiles), according to whether their distribution was Gaussian.

The questions about knowledge were gathered in 3 categories (cf. Table 1):

1. 7 questions concerning the essential measures to take in the event of simple hypertension
2. 6 questions about management of resistant or complicated hypertension
3. 7 questions about non-essential but useful general knowledge for a good management of hypertension

**Table 1:** Classification of the items of the pre-test and post-test

Codes	Items	Reponses
Essential knowledge to diagnose and treat a simple HTA: no sign of complications (simple HTA)		
CIS-1 Diagnosis	HTA if BP is 140–159 and/or 90–99 or higher than those numbers in the office	yes
CIS-1 Medical treatment	Could we stop treatment if BP became normal?	no
CIS-2 Medical treatment	Which antihypertensor should be prescribed first intention at for a patient at risk of stroke or IC to the CSB?	diuretics (thiazide)
CIS-3 Medical treatment	Among the following medicines, indicate the financially accessible ones for the hypertensive Malagasy: diuretic thiazides, beta blockers, calcium antagonists, inhibitors of converting enzymes (short-acting/captopril), antagonists of angiotensin 2 (ARA2) receptors	diuretics and inhibitors of converting enzymes (short-acting/captopril)
CIS-1 Hygiene-dietetics measurement	A reduction in overweight involves a reduction in the numbers of BP	yes
CIS-1 Follow-up	Among the following proposals on the follow-up of the HTA, select the ones that are correct: weekly during the first six months of treatment, monthly during the first six months of treatment, semi-monthly after 6 months of treatment, quarterly after 6 months of treatment.	Monthly during the first six months of the treatment and quarterly after six months of the treatment.
CIS-2 Follow-up	Should we stop the treatment of the HTA in the event of the appearance of side effects?	no
Essential knowledge for resistant hypertension—i.e., not stabilized by the drugs available in the HBC—and complicated, i.e., presenting signs of repercussions to other organs (resistant and complicated HTA)		
CIRC-1 Medical treatment	The antihypertensor is prescribed first intention at for a patient at risk of renal insufficiency with the HBC	Diuretics: furosemide, inhibitors of the converting enzymes at short-acting time(captopril)
CIRC-2 Medical treatment	Among following associations of the antihypertensor—IEC + sartan, IEC + diuretics, IEC + beat-blocking, IEC + inhibiting calcic—which are synergistic?	IEC + diuretics;IEC + inhibiting calcic
CIRC-3 Medical treatment	In a hypertensive crisis, in which case we do not recommend antihypertensive treatment in an emergency?	Vascular accident of the ischemic type
CIRC-4 Medical treatment	Among following associations of the antihypertensors Beta Blocker + Vérapamil, Beta Blocker + Dihydropyridin, Blocking alpha + Dihydropyridin, Converting enzyme Inhibitor + diuretics to save potassium, Converting enzyme Inhibitor + Vérapamil, which are disadvised?	Beta Blocker + Vérapamil; Converting enzyme Inhibitor + diuretics to save potassium
CIRC-1 hygiene-dietetics measurement	Which are the hygiene-dietetics measurements adapted to the Malagasy context: a pinch of salt with each mealto avoid food or industrial preparations rich with salt (canned, pork-butchery, sauces), stopping alcohol consumption, stopping tobacco consumption, preparing a family dish to avoid fatty meats, or drinking at least 1.5 l of water per day?	All
CIRC-1 Follow-up	What are the side effects that could appear when prescribing an inhibitor of converting enzymes?	a rise in the creatininemy, a cough
Useful Knowledge for HTA (useful HTA)		
CU-1 Diagnosis	True statement regarding the HTA: A. has a blood pressure > 140/90 mmHg is used as definition of the HTA because it is starting from this level that the risk of complication appears; B .the isolated systolic HTA is defined by a systolic pressure > 140 mmHg and a diastolic pressure < 90 mmHg; C. the curable forms of HTA account for approximately 5% of all cases of HTA; D. the most frequent complications of hypertension are those related to atherosclerosis.	b and d
CU-2 Diagnosis	The indicators of risk of HTA are age, overweight, diabetes, stress, alcohol, excessive consumption of sodium	yes
CU-3 Diagnosis	Exact proposal of the HTA: the values of reference are different at the health center or the hospital and residence	no
CU-1 Medical treatment	Which is the false statement about the HTA: A. It has a level of total cardiovascular risk incurred by hypertension that must logically lead to the decision of antihypertensor treatment; B. In the choice of a antihypertensor, the hypotensive effectiveness is additional to other properties, specific to each product; C. a hypertensive urgency is distinguished from a simple tensional push by the existence of signs of visceral suffering; D. hypertension is considered resistant when it cannot be controlled despite the association of 3 different active ingredients, including diuretics.	b
CU-2 Medical treatment	The goals of the treatment of the HTA is to normalize blood pressure numbers and to prevent the appearance of complications	yes
CU-1 Follow-up	The treatment by thiazidic diuretic justifies first-intention controls of the following parameters: kaliemy, creatininemy, clearance of creatinin, uricemy	yes
CU-1 Complication	Which can be the complications of the HTA?	Stroke, left ventricular hypertrophy, Arteriopathy of the lower extremities

Each item was coded 1 for a correct answer and 0 for an incorrect answer. Participants’ average score was calculated by dividing the total marks obtained by all participants for each training modality by the total number of participants. The comparison of the scores for the knowledge before and after the training course was carried out by a paired t-test for each training modality.

The comparison of scores for knowledge between the two training modalities and between the professional groups (doctors versus paramedics) was performed by Student’s t-test.

The comparison of participants’ satisfaction with each training modality was performed by a chi-squared test. The significance was fixed at *p* < 0.05. These analyses were performed with SPSS18 software.

## Results

### Characteristics of the participants (cf. Table 2)

Among the 101 heads of HBC working in 3 SDSP, 92 (91%) took part in the training; 48 (52%) were doctors, and 44 (48%) were nurses. The distribution of the participants in terms of number and professional group were unequal between the two training modalities.

**Table 2:** Characteristics of the participants, according to the methods of formation and of the profession

Participants	Together N	Internet VS + VD N	Correspondence DVD N
Together Heads of HBC N (%)	92	56 (61%)	36 (39%)
Age (years) Median	45 [33–52]	42 (31–52)	46 (35–52)
Minimum–Maximum	24–58	24–58	30–57
Median length of service [q1; q3]	19 (7–26)	19 (5–27)	21 (10–27)
Doctor N (%)	48	24 (50%)	24 (50%)
Age (years) Median [q1; q3]	45 (31–52)	44 (31–52)	45 (34–45)
Minimum - Maximum	28–58	26–58	30–57
Median length of service [q1; q3]	16 (2–23)	16 (2–25)	17 (6–17)
Paramedics N (%)	44	32 (72%)	12 (28%)
Age (years) Median [q1; q3]	44 (33–52)	42 (32–50)	48 (38–48)
Minimum – Maximum	24–58	24–58	30–56
Median length of service [q1; q3]	21 (10–29)	20 (6–30)	25 (15–25)

The median age of the heads of CSB was 45 years (minimum 24, maximum 58 years). The internet group was younger (42 years) than the DVD group (46 years) (*p* < 0.05). The paramedics in the DVD group were on average older (48 years) than all the other participants.

The median duration of the paramedics’ service was higher than that of the doctors’ (20 versus 16) (*p* < 0.05).

### The evolution of the knowledge of the heads of HBC according to the training modalities (internet versus DVD) per professional group and per hypertension characteristics (Table 3)

#### By training modality

Before the training, regardless of the training modality, the mean score of knowledge obtained by all participants was 7 ± 2. After training, the score was 14 ± 2.5 in the internet group (i.e., by VS or VD) and 15 ± −2.7 in the DVD group (see Table 3). There was no difference between the gain in the internet and DVD groups (*p* = 0.076).

**Table 3:** Average score and standard deviation of knowledge in pre-test and post-test according to the methods of formation (via internet and via DVD), according to the professions and the characteristics of the HTA (simple HTA, resistant or complicated HTA, useful knowledge of HTA)

Groups	Number of Participants	Average score before training (Sd^a^)	Average Score after training (Sd^a^)	Average profit	*p*-value
By training modality					
Internet	56	7 (2,5)	14 (2,5)	7	<0,001
DVD	36	7 (2.4)	15 (2,7)	8	<0,001
By profession					
Doctors Internet	24	7 (3,1)	14 (2,4)	7	<0,001
Doctors DVD	24	8 (2,3)	16 (2,7)	8	<0,001
Paramedics Internet	32	7 (2,4)	14 (2,2)	7	<0,001
Paramedics DVD	12	7 (3,2)	14 (2,7)	7	<0,001
By hypertension characteristics					
HTA simple7 items Internet	56	4 (1,25)	5 (1,07)	1	<0,001
DVD	36	4 (0,90)	5 (0,90)	1	<0,007
Complicated and resistant HTA 6 items					
Internet	56	1 (0,6)	4 (1,16)	3	<0,001
DVD	36	1 (0,7)	5 (1,1)	4	<0,001
Useful HTA 7 items					
Internet	56	2 (1,11)	5 (1,03)	3	<0,001
DVD	36	1 (0,8)	5 (1,06)	4	<0,001

*Sd* ^a^ Standard deviation

#### By profession

In the internet group, the average score after the training for the doctors and the paramedics was the same 14.

The average knowledge gain of the doctors using the internet was 7 and of those using DVDs was 8.The difference between the two training modalities was significant (*p* = 0.008).

For paramedics, the average gain was identical to +7 in the two groups (*p* = 0.364).

#### By hypertension characteristics: Simple hypertension, resistant and complicated hypertension and useful knowledge for the management of hypertension

The average gain of knowledge of the item management of simple HTA was identical for the two training modalities (+1). On the other hand, for the 6 items on resistant or complicated hypertension and the 7 items on useful knowledge for HTA, the average gains were even, 4 in the DVD group and 3 in the internet group. The difference between the two modalities was significant (*p* < 0.025).

### Participants’ acceptance of the two training modalities

The overall rate of participants’ appreciation was the same for the two training modalities (89%). It was the same for the contents of course (85% internet, 84% DVD), for the images and sound (79% internet, DVD 80%) and for the duration of the training (68% internet, 71% DVD). Slightly one third fewer (29%) training participants using the internet would agree to pay course fees, compared to 16% of those in training using DVD.

All participants in the focus group found the training sessions interesting and appreciated that they were run efficiently. Twenty four Head of HBC have participated in the focus group, 15 doctors and 9 paramedics (4 doctors and 4 paramedics for the visioconference, 5 doctors et 3 paramedics for the videoconference, 6 doctors et 2 paramedics for the DVD). Those internet training found that the duration was short and that they could not ask questions. Those in the DVD training found that the explanations were fast, and the disadvantage was that it was not possible to ask questions and request clarifications. The advantage for the DVD training was that it is possible to re-watch the course if necessary.

Despite these inconveniences, all participants (DVD and internet groups) found that the information was very clear.

### Cost analysis

The common costs of the two training modalities were 0.86 euro per participant. This included the tutor fees (0.33 euros), the duplication of the knowledge-assessment questionnaires (0,21euros) and the duplication of the training resources (0.32 euros). The cost of a 60-min internet connection was 18 euros for VS and 8 euros for VD. The production of DVD was 18 euros. Overall, the cost per participant was 4 euros for the training via VS and 3 euros for the training via VD or via DVD, meaning that for 92 rural HBC in 3 SDSP, the expenditure was 368 euros for the training via VS and 276 euros for the training via VD or DVD.

## Discussion

Following this empirical study, we can conclude that the two training modalities, via the internet and via DVD, had the same effectiveness in terms of knowledge improvement on the management of hypertension. The two methods of distance training continuing medical education allowed the update of the knowledge for the health professional in remote areas and are acceptable by healths professionals.

In both cases, the level of knowledge doubled after the training, but it did not reach 100%. The gain of the training via DVD was slightly higher (+1) than the gain of the training via the internet. There was no difference between the average scores for the knowledge acquisition of the characteristics for simple hypertension and resistant or complicated hypertension, according to the training modalities. The majority of the participants appreciated the two training modalities. They thought that both could be used as remote-delivery training to provide continuous medical training.

The training cost of using VD or DVD was 3 euros per participant, one euro less than using VS. Training via VD or DVD was less expensive than training via VS, but the advantage of VS is that it offers direct interaction with the tutor. To enable the heads of all 2504 Madagascar HBC to access the remote-delivery training, the cost of training via VD and DVD would be obviously lower than training via VS (7512 euros vs. 10.016 euros).

The limitation of this study is the absence of the randomization of the leaders of HBC.

The bias here is limited because the basic knowledge (before training) in both groups was the same (the overall score but not per group). The generalization of the data to all Malagasy rural area physicians is not possible.

This study is the first study to compare training via the internet and training via DVD.

The effectiveness of the training of health professionals via the internet or correspondence has been evaluated for approximately ten years, with a recrudescence in the number studies in the past five years. The effectiveness has mainly been measured with two indicators, knowledge acquisition gain (mean score or percentage) and satisfaction with the teaching.

Five non-comparative studies were carried out mainly in countries with low income.The topics varied widely, i.e., palliative care [13], AIDS [14], management of hypercholesterolemia [15], management of hepatitis C viral [16], support of advanced-life patients [17], research on epidemiology and biostatistics, [18] prevention of falls [19], medical ethics [20], and cancer [21]. Five 5 non-comparative studies were conducted. Two evaluated training on digital support (CD-ROM: Doctors in Tunisia [22] and France [23]), and 3 evaluated training via internet (2 videoconferences: doctors in Iraq [24], Cambodia [25] and 1 video conference: health workers from 12 African countries [26]. The first two studies aimed for a threefold and fivefold gain of knowledge and the satisfaction of the participants on the content as well as the audio-visual presentation. In the other 3, despite the absence of an objective measurement of the knowledge gain, the participants were satisfied with the content and methodologies used.

All comparative studies were carried out in countries with a high income USA [15, 16, 21, 27, 28], Spain [13], Australia [19], Japan [17], Iran [14, 18], India [20], and none were conducted in countries with a low income.

Among these 11 studies, 9 compared face-to-face training via internet training, online internet (5), via VS (2) and via VD (2) and 2 compared online training to training via correspondence, sending documents (1) or DVD (1). The comparison concerned mainly (7) online training.

The results of these 11 studies aimed for a double increase or more in the gain of knowledge and did not reach 100% after the training, whether via the internet or via correspondence. The gain of knowledge via the internet was equal to (28 vs. 30, 81 vs. 90, 15 vs. 13, 5 vs. 3, 2 vs. 4, 4 vs. 4) or greater than (5vs.2, 59 vs. 43, 22 vs. 16, 12 vs. 5) face-to-face training. The gain of knowledge was greater for training via DVD than online training (7 vs. 5).

Patients’ satisfaction of was assessed in only 7 of the 11 studies (4 online/face to face, 1 VD/face to face and 2 online/documents and DVDs). In all these studies, the rate was high (85%), regardless of the modality used. The satisfaction rate of the heads of HBC regarding the contents (80%) rejoined with the two studies [15, 21].

The lesson learned mainly from this study is that DVD’s are as good as internet distance learning method for this kind of knowledge acquisition. By taking into account the cost of training and the absence of an internet connection in the majority of the HBC based in rural and remote areas, the training by DVD is the first choice for continuous medical training for heads of HBC. In fact, the majority of them have DVD players. Taking account of the trend of the increasing internet coverage in Madagascar, the VD would be the second viable option.

Two questions are left unanswered. Regardless of the remote-delivery training modality, the acquisition of knowledge did not reach 100%. The items least known by the participants after the training were the characteristics of simple hypertension and resistant or complicated hypertension relating to medical treatment and the follow up.

The second question concerns the relationship between knowledge acquired during the training and general practice whilst seeing a patient.

These two questions will be treated later on.

## Conclusion

It can be deduced that the three training modalities made it possible to improve participants' knowledge of the management of hypertension. The average Scores of knowledge acquisition of participant and participant satisfaction were the same for the 3 training modalities. From a cost perspective, VD and DVD were cheaper than VS the following recommendations are recommended:

- If the DSPH has an Internet connection cover, the choice should be made between VD and DVD.
- If the coverage in Internet connection is weak or does not exist at the level of the DSPH, the mode by DVD is the only realizable.

## Abbreviations

ESC: European Society of Cardiology
ESH: European Society of Hypertension
HBC: Health-Based Centers
MHD: Hygiene dietetics measure
QCM: Questions with multiple choices
RDT: Remote-Delivery Training
SFC: French Society of Cardiology
VD: Videoconferencing
VS: Visio conferencing
